# Detection of Intra-Tumor Self Antigen Recognition during Melanoma Tumor Progression in Mice Using Advanced Multimode Confocal/Two Photon Microscope

**DOI:** 10.1371/journal.pone.0021214

**Published:** 2011-06-22

**Authors:** David A. Schaer, Yongbiao Li, Taha Merghoub, Gabrielle A. Rizzuto, Amos Shemesh, Adam D. Cohen, Yanyun Li, Francesca Avogadri, Ricardo Toledo-Crow, Alan N. Houghton, Jedd D. Wolchok

**Affiliations:** 1 Swim Across America Laboratory, Immunology Program, Sloan-Kettering Institute for Cancer Research, New York, New York, United States of America; 2 Department of Medicine, Memorial Sloan-Kettering Cancer Center, New York, New York, United States of America; 3 Research Engineering Lab, Sloan-Kettering Institute for Cancer Research, New York, New York, United States of America; 4 Weill Medical College of Cornell University, New York, New York, United States of America; Saint Louis University School of Medicine, United States of America

## Abstract

Determining how tumor immunity is regulated requires understanding the extent to which the anti-tumor immune response “functions” *in vivo* without therapeutic intervention. To better understand this question, we developed advanced multimodal reflectance confocal/two photon fluorescence intra-vital imaging techniques to use in combination with traditional *ex vivo* analysis of tumor specific T cells. By transferring small numbers of melanoma-specific CD8+ T cells (Pmel-1), in an attempt to mimic physiologic conditions, we found that B16 tumor growth alone was sufficient to induce naive Pmel-1 T cell proliferation and acquisition of effector phenotype. Tumor -primed Pmel-1 T cells, are capable of killing target cells in the periphery and secrete IFNγ, but are unable to mediate tumor regression. Within the tumor, Pmel-1 T cells have highly confined mobility, displaying long term interactions with tumor cells. In contrast, adoptively transferred non tumor-specific OT-I T cells show neither confined mobility, nor long term interaction with B16 tumor cells, suggesting that intra-tumor recognition of cognate self antigen by Pmel-1 T cells occurs during tumor growth. Together, these data indicate that lack of anti-tumor efficacy is not solely due to ignorance of self antigen in the tumor microenvironment but rather to active immunosuppressive influences preventing a protective immune response.

## Introduction

It is known that the immune system can generate responses to antigens expressed by cancer cells [1]. However, barriers remain at the level of the tumor, and possibly in the secondary lymphoid organs, which prevent effective cancer immunity [2], [3]. Determining the exact nature of these barriers is of the utmost importance in development of effective tumor immunotherapy.

During malignant transformation and progression, rounds of immuno-editing occur, resulting in a tumor with the ability to evade immune destruction [4]. While the concept of immuno-editing has become accepted, the exact escape mechanisms used by various cancers remain a source of debate. A growing body of evidence suggests active inhibition of T cell effector functions by tumor cells and/or cells of the tumor stroma [5], [6], [7]. However, passive processes, such as immune ignorance or deletion of highly reactive “self” T cell clones during central tolerance induction may also prevent tumor eradication [8]. The processes that avert autoimmunity may also prevent the generation of effective anti-tumor immunity [9]. While these various mechanisms have different mediators and temporal separations, they may not be mutually exclusive. It is possible that multiple processes cooperate to prevent effective tumor immunity.

Technological advances in imaging permit us to examine, through the use of two photon laser scanning microscopy (TPLSM), the anti-tumor immune response in real time, *in vivo*. TPLSM has already improved our understanding of *in vivo* priming of the immune response to foreign antigens, by examining T cell interactions in secondary lymphoid organs [10], [11]. In addition, recent research has examined effector phase anti-tumor responses to tumors expressing the foreign antigen OVA (hen ovalbumin) [12], [13], [14]. However, studies have yet to investigate the immune response to a self antigen in a progressive syngeneic tumor model. The use of artificial foreign antigen tumor models, supra-physiologic numbers of transferred T cells and explanted tumor imaging may not be representative of natural conditions. Although TPLSM imaging is inherently invasive, intra-vital techniques which preserve the integrity of the tumor microenvironment can lessen the impact of imaging on the biology. These include tracking of T cell responses against natural self antigens in a relatively un-manipulated manner using low numbers of precursors which more closely match endogenous populations.

In this study, we attempted to visualize the anti-tumor immune response in settings that more closely mimic physiological conditions, through the development of a novel high speed TPLSM with the added capacity to capture backscattered or reflected light. Combining intra-vital imaging techniques with *ex vivo* analysis, we tracked small numbers of un-manipulated self antigen tumor-specific CD8+ T cells both temporally and spatially. Naïve B16 tumor specific Pmel-1 CD8+ T cells transferred into tumor-bearing mice proliferate, acquire an activated effector phenotype, and traffic to the tumor. Within the tumor, Pmel-1 T cells have confined mobility, displaying long term interactions with tumor cells. In contrast, co-transferred non tumor-specific OT-I effector T cells showed neither confined mobility nor long term interactions with B16 tumor cells unless B16 was engineered to express OVA. These data support intra-tumor recognition of cognate self antigen by Pmel-1 T cells demonstrating that despite tumor progression, intra-tumor ignorance of self antigen does not act as a barrier to tumor immunity.

## Materials and Methods

### Ethics Statement

All mouse procedures were performed in accordance with institutional protocol guidelines at Memorial-Sloan Kettering Cancer Center (MSKCC) and were maintained according to NIH Animal Care guidelines, under a protocol 96-04-017 approved by the MSKCC Institutional Animal Care Committee.

### Mice and tumors

C57B/6J, C57B/6J^THY1.1^, GFP and CFP mice were acquired from Jackson laboratories. Pmel-1 mice, were a gift from Nicolas Restifo (NCI, MD. The B16F10 mouse melanoma cell line was originally obtained from I. Fidler (M.D. Anderson Cancer Center, Houston, TX) (B16). For imaging experiments B16 was stably transfected by electroporation with a pcDNA3.1 vector (Invitrogen) encoding EYFP (Clonetech) (YFP-B16). Cells were cultured in RPMI 1640 medium containing 7.5% FBS. Growth medium for YFP-B16 was supplemented with 0.5 mg/ml G418.

### Flow cytometry and Antibodies

Tissue was homogenized through 40 µm strainers to produce single cell suspensions for FACS analysis. Cell suspensions were labeled using the following BD antibodies CD3, CD4, CD8 CD11b, CD44, CD45, CD62L, NK1.1, GR-1, Foxp3 (Ebioscience), CFSE (Invitirogen). Samples were acquired on either 5 channel or 12 channel flow cytometers (BD).

### Adoptive transfer of CD8+ T cells

CD8+ T cells were prepared for adoptive transfer by isolation of spleen and LNs from donor mice. Tissue was homogenized and CD8+ T cells were positively selected using magnetic antibody cell separation (Miltenyi). CD8+ T cells were resuspended in PBS and transferred to recipient mice through tail vein injection.

### *In vivo* killing assay

Congenic CD45.1 splenocyte targets were loaded with 20 µg/ml of either relevant (gp100) or irrelevant (OVA) peptides in media for 1 hour at 37° and then labeled either a high (5 µM) or low (0.5 µM) level of CFSE, respectively. 5×10^5^ of each target cell was then transferred by tail vein injection to recipient mice at a 1∶1 ratio. Measuring the ratio of gp100 loaded targets to OVA loaded targets isolated from the CD45.1 gate in the control mice which did not receive tumor provides the baseline level of gp100 target cells which remain after transfer.

### Intravital Imaging

YFP-B16 tumors were injected in the left flank of mice upstream of the inguinal LN. 6−10 days post AT (days 9−13 of tumor growth), 2 mice were imaged per day to detect the peak of Pmel-1 T cell tumor infiltration per experiment, (usually 7−8 days post AT, day 10−11 of tumor growth). Multiple time points were imaged in order to find the peak point of infiltration and compensate for variability associated with each tumor injections, priming response and 3 dimensional tumor structures. Mice are anesthetized with 1.5% isoflurane delivered by 1 L/hour O2 on a heated platform maintained at 37°C. Surgery is performed to open up a skin flap to expose the tumor and inguinal LN while maintaining vasculature integrity. The tumor and TDLN are then isolated under nylon washer mounted coverslips, with PBS and visualized with a heated (37°C) objective. Temperature of isolated tissues is checked with a thermal probe to ensure it is maintained at 37°C. Time lapse images are acquired with a Z-depth on the average of 100−150 µm with 3 µm between steps, starting at +/− 10 µm from the top edge of the tumor. Mosaic images are taken with 50 µm overlaps between adjacent regions. The video capture rate of over 20fps enables 6∶1 frame averaging with a sample area that includes up to 9 adjacent 270 µm×270 µm×100 µm volumes to produce a mosaic image every 80–120 seconds. Time lapse images vary in length from 60–120 min. To control for tissue viability after surgery and as a baseline for Pmel-1 T cell mobility, inguinal TDLNs were imaged alongside tumors in each experiment.

### Image Analysis

Mosaic Images compiled together using custom MatLab program and imported into Volocity. Movies were generated to determine if regions imaged displayed viable cell movement (Blood flow, YFP ghosts) before T cell tracking was performed on individual regions. Images were corrected for contrast with 3×3×3 pixel noise filtering to remove background signal where necessary. Individual T cell tracks were calculated using Volocity automatic object acquisition and tracking modules. Each track was verified for algorithmic errors. Image drift was calculated by tracking 3–6 tumor landmarks per image, per time point during time lapse. The average movement of the landmarks was removed from all calculated trajectory and velocity measurements accordingly. Intra-tumor T cells positions were calculated by producing a high digital threshold map of the tumor images were analyzed using and custom-developed Matlab code. Mean distance from the Central Point of Movement (Mean DCPM) was calculated using the centroid positions of each cell tracked for over 10 time points according to this equation: 
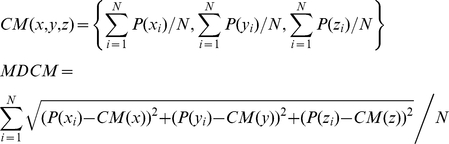



P(x), P(y) and P(z) are the Cartesian coordinates of individual observations. N is the length of observations. CM is the Cartesian coordinate of the Central Point of Movement. Mean DCPM is the mean distance of individual observation from CM. Statistical comparisons of Pmel-1 vs OT-I were done in Graph pad Prism 5 using a student T test. Statistical comparison of the raw mean DCPM was done using Kruskal-Wallis test with pair wise comparisons. Mean DCPM were then converted to mm Log10 to enable population frequency distribution calculations, in order to determine the mode for each population. Due to high density of YFP-B16 tumor mass, images and movies are displayed in 3D volumes instead of 2D projections to enable more accurate presentation of cell location respective to other.

## Results

### Peak effector and regulatory immune cell tumor infiltration occurs 8**–**10 days after B16 tumor challenge

**Figure 1:**
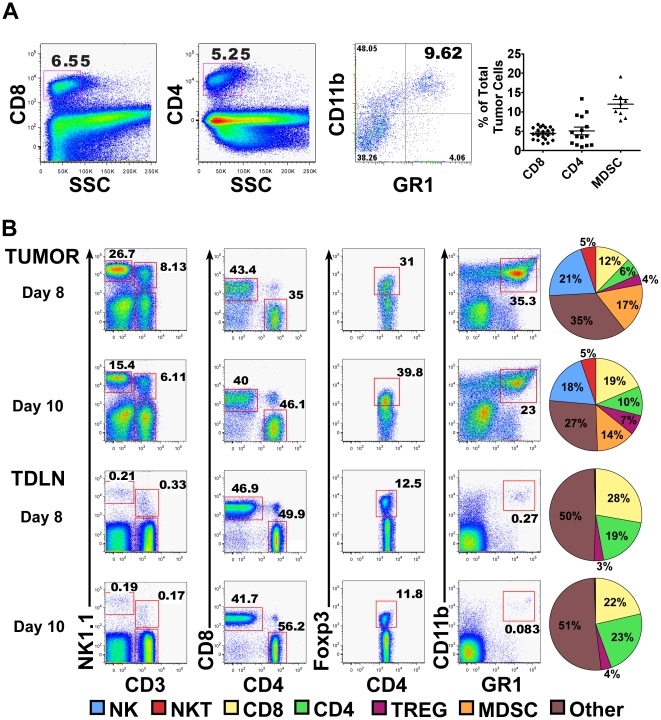
Effector and inhibitory immune cell infiltrates in B16 tumors with parallel kinetics. 2.5×105 B16 tumor cells were suspended in matrigel and inoculated into WT C57B/6 mice. Tumors and TDLN were isolated and assessed for immune cell percentages to determine the peak of tumor infiltration time. (A) Representative FACS plots show CD4+, CD8+ and MDSC (GR1+/CD11b+) percentages of total tumor cells isolated from individual 8 day old B16 tumors. Graphs show mean (+/−SEM) infiltration for CD8+ (4.4%, N = 22) CD4+ (5%, N = 15) and GR1+ CD11b+ (12%, N = 9) in day 8–10 B16 tumors. (B) FACS plots of leukocyte marker staining, post density gradient centrifugation of total tumor and TDLNs. NK1.1, CD8, and CD11b plots were gated on live cell fractions (7AAD-). Parent gate for MDSCs (CD11b+GR1+) plot is CD3-NK1.1-. Parent gate for CD4+/CD8+ T cells is CD3+NK1. N = 4 and 15 for day 8 and day 10, respectively. Pie charts show distribution of cell types as a percentage of the leukocyte compartment inside the tumor.

In order to investigate the intra-tumor dynamics of self antigen responses during the peak of the anti-tumor immune response, we first determined the precise activation kinetics of the anti-B16 immune response. Prior research, by our group and others, has been limited to examining tumor infiltrating lymphocytes during late stages of tumor growth (greater than 14 days after tumor challenge), or during immunotherapy [2], [15]. Therefore, we began our analysis 2 days after tumor challenge, using tumors injected subcutaneously in growth factor reduced Matrigel, to develop a more complete understanding of the early tumor microenvironment [16]. Using this method, we determined that peak immune infiltration (as a percentage of total cells) was between days 8–10 of tumor growth (Fig 1A, B). At the peak (day 8–10), there were relatively equal percentages of CD8+ and CD4+ T cells in the tumor (∼5%). At the same time, putative MDSCs (CD11b+, GR1+ of the macrophage lineage F4/80+) constituted ∼12% of the total tumor burden. A more detailed analysis of the leukocyte/myeloid infiltrate was accomplished after density gradient centrifugation of the tumors (Fig. 1B). ∼40% of the CD4+ cells present in the tumor were Foxp3+ Tregs, demonstrating a selective recruitment of inhibitory cells to the tumor as a portion of total leukocytes compared to the TDLN. This suggests that the effector response is activated with the same kinetics as a putative inhibitory response. As tumors progressed, B16 cells composed the majority of the tumor (∼80–90% of all cells after ∼15–18+ days) and the levels of all infiltrating immune cells decreased (data not shown).

### Determining activation kinetics of T cell responses to B16 self antigen gp100

**Figure 2:**
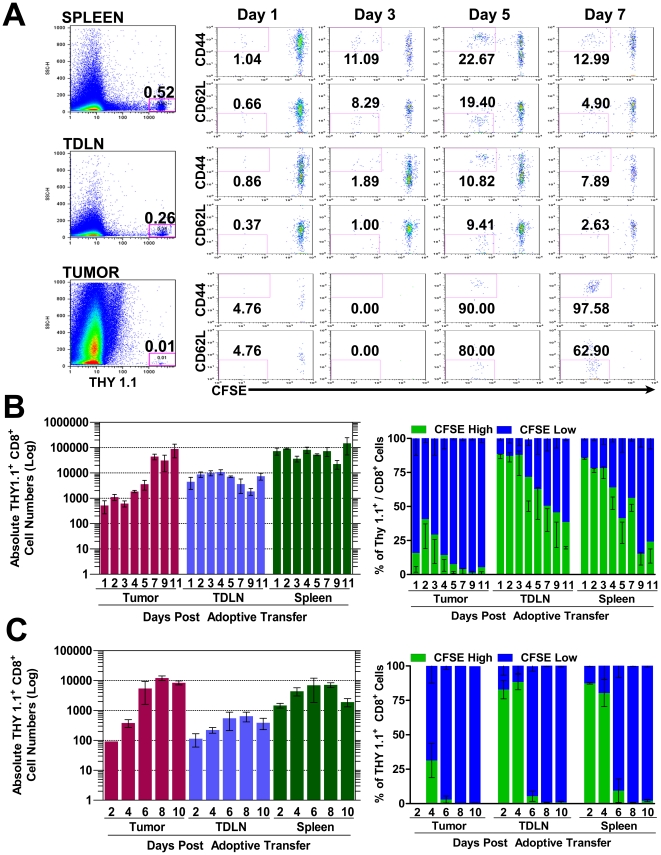
Naïve B16 specific Pmel-1 T cells are primed by tumor growth and infiltrate tumors after adoptive transfer into tumor bearing mice. 2.5×10^5^ B16 tumor cells were inoculated in matrigel into WT C57B/6 mice. Naive CFSE labeled Thy 1.1+ CD8+ Pmel-1 T cells were AT either 6 days (A, B) or 3 days (C) after tumor challenge. Spleens, TDLNs and tumors were isolated from 3 mice every 24–48 hours after AT for up to 11 days post transfer and analyzed for Pmel-1 T cell infiltration and activation (A) LEFT, representative SSC/Thy 1.1 FACS plots from 7 days post AT of 2×10^6^ naïve Pmel-1 T cells show percentages of infiltrating Thy 1.1+ Pmel-1 T cells. RIGHT panels shows CD44/CFSE, CD62L/CFSE on noted days post AT. Gates show representative percentages of activated (CD44^High^/CFSE^Low^, or CD62L^Low^/CFSE^Low^) Pmel-1 T cells infiltrating tissues at each time point. (B, C) Mean +/− SEM absolute numbers (left) and CFSE phenotype percentages (right) calculated from the SSC^low^ Thy1.1+. gate. (B) AT of 2×10^6^ CD8+ Pmel-1 T cells (C) AT of 3×10^5^ CD8+ Pmel-1 T cells Percentages shown are representative of 2–3 independent experiments with 3 mice/timepoint.

Subsequently, the rate at which B16-specific CD8+ T cells from the Pmel-1 mouse are naturally primed and traffic to the tumor was investigated. CD8+ T cells in this mouse express a naturally selected TCR and are reactive to the melanoma self antigen gp100, but are unable to reject B16 tumors without other immune manipulation [17], [18], [19]. This suggests that at the steady state, Pmel-1 T cells are either susceptible to tumor mediated inhibition or the strength of their effector function is not sufficient to provide tumor killing. We began by determining the Pmel-1 T cell response kinetics to B16 growth. Naïve Pmel-1 T cells proliferated and acquired effector phenotypes with similar kinetics when 2×10^6^ cells were adoptively transferred (AT) into mice bearing 0, 3, 6 or 9 day old B16 tumors (example of this data is shown in Fig. 2A,B). Naïve (CD62L^High^,CD44^Low^,CFSE^High^) Pmel-1 T cells infiltrated the tumor-draining lymph node (TDLN) and spleen within 24 hours (∼10,000 cells in TDLN & 100,000 in spleen), while few cells were seen in the tumor (<∼500 cells) (Fig. 2A,B). Initial activation and proliferation of the Pmel-1 T cells required three days as measured by up-regulation of CD44 and dilution of CFSE. After 5 days, activated Pmel-1 T cells (CD62^Low^,CD44^High^,CFSE^low^) began infiltrating the tumor with infiltration peaking 7 days post AT and over 90% of Pmel-1 T cells displaying an activated phenotype (Fig. 2A). Similar to the activation kinetics, Pmel-1 T cell tumor infiltration was comparable after transfer into mice 3, 6, and 9 days after tumor challenge (Sup Fig. S1A). While gp100 is expressed by normal melanocytes in C57BL/6 mice, priming of Pmel-1 T cells is tumor growth-dependent as they do not proliferate nor become activated in the absence of or during mock (Matrigel alone)tumor challenge (Sup Fig. S1B).

The transfer of supra-physiologic numbers of T cell precursors has been shown to alter responses due to competition for antigen by T cells [18], [20], so we compared AT of 2×10^6^, 1×10^6^, or 3×10^5^ Pmel-1 T cells into mice bearing 3-day old tumors. In agreement with previous studies, our data demonstrated that lower numbers of transferred Pmel-1 T cells results in greater proliferation and activation (Fig. 2C). Transfer of 3×10^5^ cells was chosen as the lower limit as it enabled detection of sufficient numbers of cells during intra-vital imaging, while remaining close to the optimal number of Pmel-1 T cells as determined in our previous adoptive cell therapy experiments [18].

### Naïve Pmel-1 T cells primed by tumor growth produce competent CTL effector T cells

**Figure 3:**
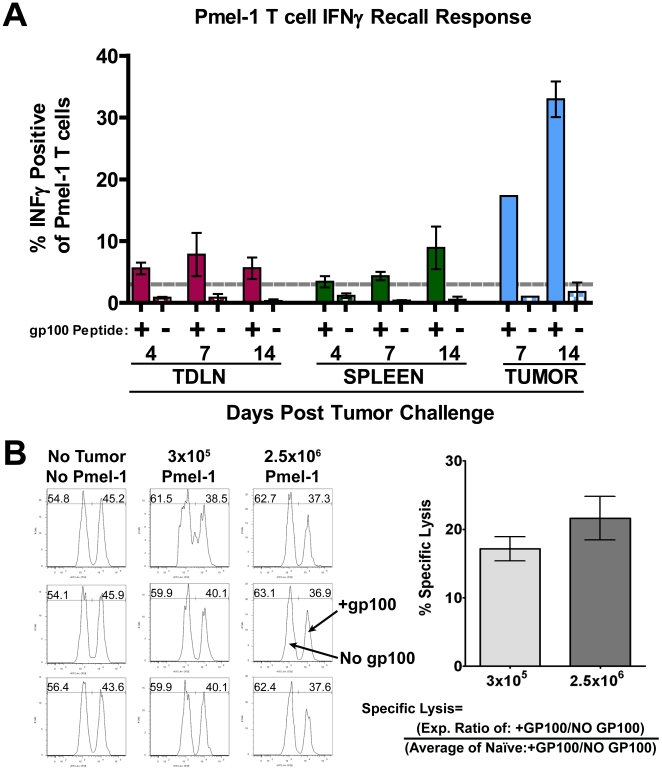
Adoptively transferred Pmel-1 T cells display lytic capability in the periphery after priming by tumor growth. A) Thy 1.1+ CD8+ Pmel-1 T cells were AT into C57B/6 mice one day prior to inoculation with 2.5×10^5^ B16 tumor cells in Matrigel. Lymphocytes were isolated from TDLN, spleen and tumor on indicated days and re-stimulated for 6 hours with irradiated, gp100_25–33_ peptide-pulsed EL4 cells to measure IFNγ recall (n = 3/group). Background IFNγ production was <1% (dashed line). (B) Six days post AT of 3×10^5^ naive Thy 1.1+ CD8+ Pmel-1 T cells (day 9 of tumor growth). Congenic CD45.1 splenocyte targets, differentially labeled with CFSE and loaded with either relevant (gp100, CFSE^HIGH^) or irrelevant (OVA, CFSE^LOW^) peptides were AT into recipient mice at a ∼1∶1 ratio. One day later, spleens were isolated and FACS analysis was preformed. Histogram plots show CFSE levels from the CD45.1/SSC gate. Graph shows mean and SEM for specific lysis calculated using formula shown (n = 3/group).

Since naive Pmel-1 T cells proliferate and acquire an effector phenotype solely in the presence of B16 tumors, it suggests that there is no systematic defect in physiological antigen recognition. However, we could not exclude the possibility that priming is somehow defective. This has been observed in CT44 tumors expressing influenza HA antigen where tumor specific CTL primed in the presence of antigen specific Tregs appear fully activated in the TDLN, but have reduced effector capacity in the periphery, failing to cause tumor regression [21], [22]. Therefore, we assessed the quality of the Pmel-1 effector response by examining cytokine production and killing of cognate targets. Starting as early as 4 days after tumor challenge, we found that ∼5% of Pmel-1 T cells have detectable levels of IFNγ in the TDLN (Fig. 3A). By seven days after tumor challenge, a large percentage of tumor infiltrating Pmel-1 T cells (∼17%) are IFNγ positive, increasing to over 30% by day 14. This data is in agreement with the activation kinetics described above, as tumor infiltrating Pmel-1 demonstrated the largest percentage of effector cells compared to TDLN and spleen.

Although physiologic priming of Pmel-1 T cells is sufficient to produce effector cytokines, tumors grow progressively, suggesting that Pmel-1 T cells are unable to kill inside the tumor. However, if immune inhibition only occurs in the tumor, it is possible that Pmel-1 T cells could display lytic ability in the periphery. To establish if tumor-primed Pmel-1 T cells have anti-gp100 lytic potential, we performed an *in vivo* killing assay. Target cells, loaded with either the gp100 peptide or an irrelevant peptide (OVA), were injected six days post Pmel-1 AT to coincide with the peak of Pmel-1 T cell priming. Examining changes in gp100/OVA target ratio shows that Pmel-1 T cells have lytic ability in the periphery (Fig. 3B). Even though Pmel-1 effector T cells represent only ∼0.1 or 0.5% of the spleen at day 7 based on transfers of 3×10^5^ or 2.5×10^6^ naive cells, we were able to detect ∼17% and 21% specific lysis of gp100 loaded targets, respectively (Fig. 3B). Together, the fact that Pmel-1 T cells can recall IFNγ and display detectable lytic capacity implies that the priming received in the TDLN is not inherently defective. This would suggest that the response to self antigen may be regulated in the tumor.

### Unique dual mode reflectance confocal and two-photon microscope permits video-rate imaging of fluorescence-labeled and un-labeled cells

**Figure 4:**
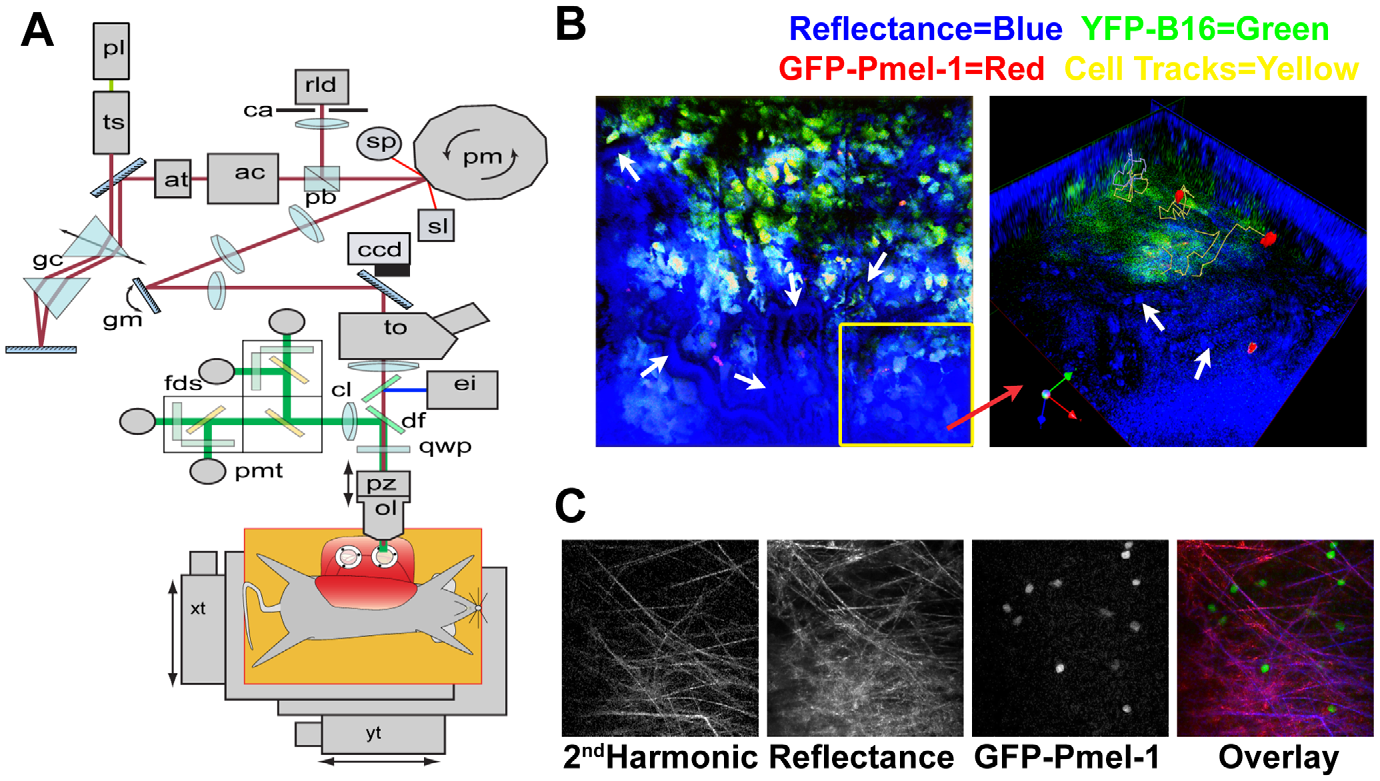
Custom two-photon microscope provides advanced reflectance capture capabilities. (A) The beam from a titanium-sapphire laser (ts) passes through a polarizing beamsplitter (pb) and on to a rotating polygonal mirror (pm) driving the fast scanning axis. The beam is relayed to a galvanometer mirror (gm) providing the slow scanning axis. Together, the scanners generate a frame rate of 22 frames per second (fps) at 512×512 pixels. The fluorescence signal generated in the tissue is reflected towards four photomultiplier tubes (pmt) separated by dichroic filters (df) to emitter filters for CFP, GFP, YFP, and a red marker (propidium iodide). Backscattered 910 nm light is also collected by the objective lens, retracing the optical path and collected through the confocal detector aperture (ca) onto the avalanche photodiode (rld). Depth scanning is provided by a piezoelectric translator (pz) that positions the objective lens (ol). A programmable XY motorized stage (xt, yt) is used to form tiled composite images. A full detailed description is available in Sup Fig. S3. (B) (LEFT) White arrows in left image demonstrate the location of blood vessels detected in the reflectance; insert yellow box (Right) is 3D composite image. (RIGHT) Reflectance also can detect unlabeled cells, such as red blood cells shown by white arrows. (C) B&W panels show: Collagen fibers detected through second harmonic generation, reflectance imaging and GFP-Pmel-1 T cells. Pseudo-color overlay (RIGHT) shows co-localization of collagen (blue), with reflectance (red), Pmel-1 T cells are shown in green.

Having established the early priming kinetics of the immune system against B16 tumor growth, we next developed a system to investigate intra-tumor dynamics of the response. To enable intra-vital imaging in conditions that most closely mimicked the physiologic environment, we engineered a novel high speed dual mode, reflectance confocal and TPLSM (Fig. 4A). Combining a high frame rate (∼22fps) with a programmable stage, our microscope permits imaging of multiple adjacent regions continuously. This important feature enhanced our ability to track *in-vivo* activated Pmel-1 T cells as they represented only <∼0.01% of the total tumor, allowing us to observe larger imaging volumes producing a more representative sample of the tumor infiltrating Pmel-1 T cells.

Our design achieves high scanning rates with no beam retrace exposure; resulting in short laser dwell times (∼120 ns) and lower levels of phototoxicity. This is achieved by combining a 32–facet polygon mirror spinning at approximately 22,000 RPM for the fast, horizontal (X-) scanning with galvanometer generated vertical (Y-) scanning (Fig. 4A & Sup Fig. S2 for detailed description). The unidirectional rotation of the polygon mirror eliminates retrace laser exposure of the sample and the fast spin rate allows this scanner to reach up to 22 fps.

Additionally, we included the capacity of collecting backscattered infrared laser light reflected from tissue samples, along with four fluorescence detector channels. Reflectance imaging provides much needed context to the “black void” usually seen in other two photon images, producing a contrast picture of the tissue being illuminated (Fig. 4B, C). While commercial systems discard the reflected infrared light, in our system it retraces the beam path, and is collected with a confocal detector (Fig. 4A, Sup Fig. S2). This enables visualization of extracellular matrix collagen fibers (similar to the 2^nd^ harmonic optical signals generated by collagen fibers [23]) along with unlabeled cells (Fig. 4B, C). Reflectance also permits the analysis of T cell migration in the blood vessels as well and identification of flow rates of red blood cell (Fig. 4C, Movie S1). This alleviates the need for marker dyes to measure the location of vasculature, or second harmonic generation to visualize collagen fibers, freeing up fluorescent channels for biological measurements.

### Development of B16 Intra-Vital Imaging Model

In order to assay if Pmel-1 T cells function inside progressing B16 tumors, Pmel-1 transgenic mice were crossed with transgenic mice expressing green fluorescent or cyan fluorescent protein (referred to as GFP-Pmel-1 & CFP-Pmel-1, respectively) and B16 was transfected with enhanced yellow fluorescent protein (B16-YFP). Additionally, as a control, non specific OT-I T cells were crossed to GFP mice, and B16 which expresses OVA (MO4) was transfected with YFP (MO4-YFP). Importantly, activation and tumor infiltration of transferred double transgenic fluorescent Pmel-1 cells was comparable to non-fluorescent single transgenic pmel-1 cells (data not shown). Similarly, B16-YFP chosen for experiments grew with comparable kinetics and had similar immune infiltration to the parental B16, remaining a target for gp100 specific lysis by activated Pmel-1 T cells (Supplemental Fig. S3A, B).

### Pmel-1 T cells engage in long term interactions with tumor cells demonstrating intra-tumor self antigen recognition

**Figure 5:**
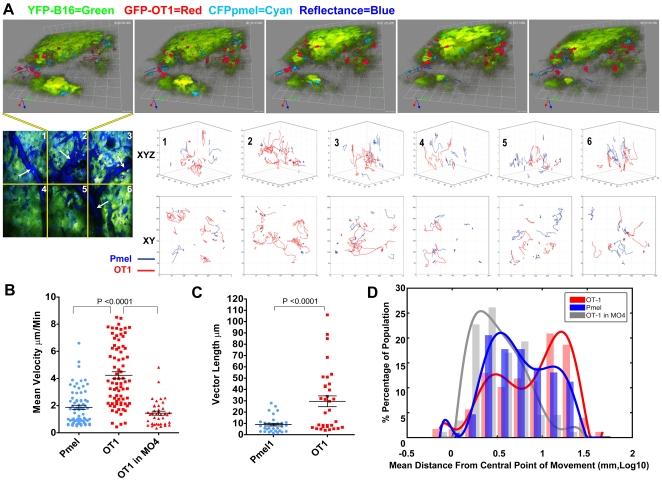
Intra-tumor mobility of Pmel-1 T cells is antigen specific. 1×10^5^ YFP-B16 tumor cells were injected in matrigel into WT C57B/6 mice with AT 3 days later of 3×10^5^ naive CD8+ CFP-Pmel-1, and 3×10^5^ naive GFP-OT-I T cells were AT into WT C57B/6 mice bearing 3 days old YFP-B16 tumors. 4 days later of 2.5×10^4^ LPS stimulated SIINFEKL loaded DCs injected into the contra-lateral footpad. (A) Representative image sequence from the 2nd region (top panel) of a 6 region time lapse image (bottom left image). Due to high density of YFP-B16 tumor mass, images and movies are displayed in 3D volumes instead of 2D projections to enable more accurate presentation of cell location respective to other. Likewise, reflectance data has been removed from top images in order to better facility presentation of fluorescence imagery. Ten min and 42sec separate each frame. Bottom; overlays of Pmel-1 (blue) and OT-I (red) T cell tracks, measured for at least 10 time points, corrected for image drift are shown for each region of the mosaic image. Top row shows 3D XYZ orientation, bottom row shows birds eye XY view. (B–D) Comparisons of mean velocity (B), 10 step vector length (C), mean distance from center point of movement (DCPM) (D) plotting each individual Pmel-1 and OT-I T cells tracked for at least 5 time points. Mean and SEM are shown for B and C, with a best fit curve over histogram of the population distribution show in D with modes of the populations being 0.4 mm, 0.1 mm and 0.4 mm in Log10 for Pmel-1 T cells, OT-1 T cells and OT-1 T cells in MO4 tumor respectively. N = 2 individual experiments, with 4 mice included in the data.

Our evidence thus far suggests that Pmel-1 effector phase function is inhibited after tumor entry. Aside from active inhibition by Tregs, it is possible that the tumor micro-environment is caustic to effector T cell function through tryptophan and arginine metabolites or hypoxic conditions [24], [25]. Therefore, to determine the extent to which Pmel-1 T cells were functional, we assessed their mobility after tumor infiltration, comparing it to co-transferred non specific OT-I T cells in the same tumor. Because the overall 3D tumor structure, cytokine milieu, and/or density of collagen may affect T cell migratory patterns [26], [27], this method allows us to separate the contribution of antigen specificity from general non-specific effects of the tumor environment. Equivalent numbers of GFP-OT-I T cells and CFP-Pmel-1 T cells were AT into mice bearing 3 day B16-YFP tumors. OT-I T cells were primed in-vivo with OVA loaded DCs which were transferred 1 day later. DCs were injected into the contralateral foot pad to prevent possible interference with Pmel-1 priming in the TDLNs. This method resulted in the concurrent recruitment at the peak of tumor immune infiltration of similar numbers of activated GFP-OT-I T cells and activated Pmel-1 T cells to the tumor (Fig. 5A). Tumor regions were then chosen for imaging based on both the presence of Pmel-1 and OT-IT cells, vasculature with circulating cells and evidence of active movement by unlabeled stromal cells (detected as YFP ghosts, Movie S2). Consistent with the distribution of CD8 T cells and other leukocytes, the activated Pmel-1 T cells were found in proximity to tumor neo-vasculature (∼60–100 µm) and not more deeply imbedded in the tumor mass (Sup Fig. S4 A,B).

Initial observations showed that Pmel-1 T cells were alive, displaying highly confined mobility, and engaging in long term interactions with tumor cells (Fig. 5A, Movie S3, S4, S5; Images and movies are displayed in 3D volumes instead of 2D projections to enable accurate presentation of cell location respective to other). Notably, tumor specific Pmel-1 T cell mobility was dramatically different from the non-specific OT-I T cells which displayed no obvious long term interactions with tumor cells (Fig. 5A, Movie S4, S5). Tumor specific Pmel-1 T cells had an average mean velocity less than half the speed of non specific OT-I T cells (2.0 µm/min vs. 4.2 µm/min). In addition, the mean overall displacement of OT-I T cells was over 3 times that of Pmel-1 T cells (28 µm vs 8.9 µm) (5B,C). Comparison of OT-I and Pmel-1 tracks showed that the trajectories of Pmel-1 T cells were much more confined than OT-I T cells (Fig. 5A). To quantify these differences we measured the total volume of tumor migrated over time for Pmel-1 and OT-I T cells, as represented by the mean distance from the central point of movement (DCPM, see Methods). Comparison of mean DCPM showed that the bulk of OT-I T cells, or the mode of the population distribution, have a mean DCPM over time increased >200% compared to the bulk of Pmel-1 T cells (1.1 mm log10 vs 0.4 mm Log 10, Fig. 5D), correlating with the differences observed examining individual T cell tracks. Additionally, the differences in raw mean DCPM of the total populations were shown to be significantly different OT-I vs Pmel-1 T cells (10.9 mm vs 7.5 mm p<0.01). Even though Pmel-1 and OT-I T cells differ in mean velocity and mean DCPM, the cells have comparable distribution throughout the tumor. Pmel-1 and OT-I T cells were located 17.6 and 16.8 µm away from “non tumor regions”, inside the tumor mass, respectively (Sup Fig. S4C). Instantaneous velocities and tumor location comparisons for every data point demonstrated an equal distribution throughout the tumor for each cell type and showed no correlation between instantaneous velocity and tumor location for either the Pmel-1 or OT-I T cells (Sup Fig. S4C). This shows that Pmel-1 T cells are largely in contact with tumor cells after tumor infiltration and suggests that there is no restriction in movement through the tumor relating to antigen specificity.

To confirm that the differences between OT-I and Pmel-1 T cell mobility results from antigen specificity we examined OT-I T cell mobility in a B16 tumor which had been engineered to express OVA (MO4-YFP). Mean velocity of OT-I T cells in MO4-YFP tumor was comparable and not significantly different than Pmel-1 T cells (mean 1.4 µm/min vs 2.0 µm/min, Fig. 5B, Movie S6). Although the raw mean DCPM of Pmel-1 T cells and OT-1 T cells in MO4 tumors was different (7.5 mm vs 4.1 p<0.01), the distribution of mean DCPM OT-I T cells in MO4 is similar to Pmel-1 T cells as is shown in figure Fig. 5D with the modes of each population being the same (0.4 mm Log 10). However, OT-I T cell mean velocity and mean DCPM in OVA-expressing MO4-YFP tumor was significantly different from OT-I T cells in antigenically irrelevant B16-YFP, similar to what has been described previously for OT-I T cells in antigenically relevant and irrelevant tumors [12], [13], [14]. Overall, these data provide an important example of differences between antigen-specific and non-specific mobility in B16 tumors. Taken together, the data imply that the slower velocity, confined mobility, and long term interactions with tumor cells of Pmel-1 T cells is antigen dependent.

## Discussion

Through the combination of ex-vivo and in-vivo analysis, we have shown that the immune system is capable of generating an anti-tumor response which is able to engage in intra-tumor recognition of cognate self tumor antigen. This demonstrates that ignorance of self antigen does not function as an immune escape mechanism in this model of melanoma.

Ex-vivo analysis shows that at the peak of the anti-melanoma effector response, melanoma specific Pmel-1 T cells are primed into competent effector cells (shown by surface phenotype, IFNγ secretion and lytic capacity in the periphery) but are unable to mediate tumor regression. In fact, tumor cell killing was not detected in the regions imaged either through tumor cell disintegration during imaging or through the use of propidium iodide (Sup Fig. S4D). We have previously shown that adoptive transfer of 1/3 as many Pmel-1 T cells along with combinatorial immunotherapy results in Pmel-1 T cell dependent regression of established tumors [18]. Thus, even though Pmel-1 T cells can actually reject B16 tumors during therapeutic intervention, in un-modulated conditions, Pmel-1 effector phase function appears to be abrogated in the tumor environment. This concept is in agreement with reports demonstrating that tumor infiltration induces a transient, but reversible block in proximal T cell receptor signaling in the MCA38 tumor model [5], [28], [29]. These papers also demonstrate that although there is a proximal block in signaling, TIL do in fact demonstrate initiation of the signaling before activation of feedback inhibition loops in the TCR cascade. Therefore, our data would suggest that although the cytolytic ability of is inhibited inside the tumor, there are still sufficient levels of TCR signaling to provide stop signals and intergrin activation to the migrating Pmel-1 T cells. However, the extent of TCR signaling and synapse formation that occurs after tumor infiltration requires further investigation.

Using intravital TPLSM imaging we examined the extent to which tumor primed T cells were functional following infiltration of cognate tumor. While previous reports have examined T cell responses inside regressing tumors, these systems were limited in their ability to study tumor induced immune dysfunction [12], [13], [14]. These studies used 1) artificial foreign antigen tumor models, 2) supra-physiologic numbers of transferred T cells and 3) explanted tumor imaging. The use of OVA ignores the contribution of thymic selection to the T cell repertoire's capacity to respond to natural tumor antigens. In addition, unrealistic T cell precursor frequencies adversely modulate the subsequent response to antigen [18]. Lastly, explanting the tumor dramatically alters the *in vivo* tumor environment, changing conditions which may be directly involved in the inhibition such as hypoxia, and glucose or arginine metabolism [6], [24], [25]. For this reason, our intravital methods left the subcutaneous visceral membrane intact, preserving tumor circulation, keeping imaging conditions as close as possible to the pre-imaging *in vivo* conditions of the tumor. Combined with transfer of 50-fold fewer self tumor antigen specific T cells, it makes this research uniquely relevant to understanding the natural barriers to cancer immunity.

The ability to image in these conditions was made possible through the development of a dual mode reflectance confocal and two-photon microscope. Collection of backscattered or reflected infrared laser light during imaging provided important internal control data by illuminating the “black void” and, combined with the video rate acquisition, makes it exceedingly easy to spatially locate regions for imaging. Speed also permitted the collection of multiple adjacent regions (up to 9 with 30 slices in under 2 min) enabling acquisition of a large sample of data without extending the time the mouse remained under anesthesia with exposed tissue.

Regardless of the method of imaging used, previous reports of tumor imaging have laid the groundwork for our understanding of T cell function in progressing syngeneic tumors [12], [13], [14]. The differences in mean velocity, mean DCPM and track comparisons strongly indicate that Pmel-1 mobility in the tumor is antigen specific. This is further supported by the fact that differences in OT-I mobility in irrelevant YFP-B16 and OVA-expressing MO4-YFP mirror those seen for OT-I mobility in irrelevant antigen expressing tumors (EL.4) compared to cognate antigen OVA positive tumors (EG.7) [12], [13], [14]. Additionally, Pmel-1 T cell interactions with tumor cells have a polarized nature, looking synonymous with what is seen between CTL and target cells in-vitro (Movie S3). More research is needed to confirm the polarity of intra-tumor Pmel-1 T cells, however, combined with the data presented here, it indicates that the mobility of Pmel-1 T cells results from self antigen recognition in the tumor.

The inclusion of OT-I T cells in the experiment has also enhanced our understanding of the mechanism of intra-tumor immune inhibition. If Pmel-1 T cell mobility had been more similar to that of OT-I T cells, it would have suggested that Pmel-1 T cells, due to priming or inhibition, were ignorant of intra-tumor self antigen recognition. Moreover, had both cell types displayed a more immobile and non-active (rounded) phenotype, we might consider that a global inhibition generated by soluble factors affected all T cells within the tumor microenvironment. Yet, the evidence presented here implies that this is not the case.

A clue to how this antigen specific block may be induced comes from examining response kinetics of the entire immune infiltrate. At the point at which we imaged, when the number of anti-tumor effector cells peaks as a percentage of the tumor, the number of regulatory immune cells do as well (Fig. 1). Thus, there is a specific enrichment of regulatory cells (Tregs, MDSC) within the tumor, suggesting that this may be part of the mechanism involved in suppressing intra-tumor T cell effector function. Although the exact role these cells play, and the mechanisms involved needs to be elucidated possible clues come from studies of CT44 tumors expressing influenza antigen HA. In this model, tumor specific CD8+ T cells appear to be fully primed but are inhibited in the periphery from effector functions by antigen specific Tregs. While there does not appear to be a block in peripheral effector functions in our melanoma model, the intra-tumor Pmel-1 T cell mobility phenotype is similar to interaction between T cells and HA loaded B cell targets in the presence of antigen-specific Tregs from CT44 TDLNs [21]. However, whether or not Tregs modulate intra-tumor Pmel-1 function in a similar manner requires further investigation.

Understanding the exact mechanism and source of tumor induced immune inhibition is paramount to the design of more effective cancer immunotherapy. The intra-vital imaging techniques we employed permitted us to investigate the immune response to self antigen in a manner which could not have been accomplished through traditional *ex vivo* analysis alone. Moreover, the advances made by our microscope design can be integrated existing platforms to study various biological questions. This research demonstrates that antigen recognition can occur inside the tumor based on self proteins. Therefore, we suggest that therapeutic efforts should focus as much on determination and neutralization of the natural barriers to an anti-tumor immune response, as they currently do on priming it.

## Supporting Information

Figure S1 **Adoptively transferred Pmel-1 T cells do not prime in the absence of tumor.** A) 3×10^5^ Naive CFSE labeled Thy 1.1+ CD8+ Pmel-1 T cells were AT into B16 tumor bearing C57BL/6 mice 3, 6 or 9 day after tumor challenge. Tumors were assessed for Pmel-1 T cell infiltration 7 and 9 days after transfer, corresponding with day 10–12, 13–15, and 16–18 after tumor challenge for the three groups. No significant differences were seen in the absolute number of Pmel-1 T cells infiltrating the tumor when comparing all groups (One way anova, tukey multiple comparison). B)_1×10^6^ Naive CFSE labeled Thy 1.1+ CD8+ Pmel-1 T cells were AT into naïve C57BL/6 mice. Spleens and Lymph nodes were isolated and single cell suspensions were prepared from 3 mice every 48 hours beginning at 24 hours after transfer for up to 7 days. Samples were then analyzed to determine the extent of Pmel-1 T cell infiltration and the activation state of the infiltrating cells. (LEFT) SSC/Thy 1.1 FACS plots are representative from 7 days post transfer. Percentages of tissue infiltrating Thy 1.1+ Pmel-1 T cells are shown in the gate. RIGHT panels show CD44/CFSE on noted days post transfer in Spleen (top) and Lymph node (bottom). Gates show representative percentages of activated (CD44^High^/CFSE^Low^) Pmel-1 T cells infiltrating tissues at each time point from the parent SSC^low^ Thy 1.1+gate. Example is representative of 2 independent experiments.
(PDF)

Figure S2 **Schematic Blueprints for Dual mode cofocal/two photon microscope.** Complete list of abbreviations form figure 3A: (ac) autocorrelator, (at) attenuator, (ccd) charge-coupled device, (cl) collection lens, (ei) Epifluorescence illumination, (fds) fluorescence detection system, (gc) group-velocity dispersion compensator, (pl) pump laser, (qwp) quarter-wave plate, (sl) synchronization diode laser, (sp) synchronization photodetector (to) trinocular. As illustrated in Figure 3A, a mode locked titanium-sapphire femtosecond laser resonator, pumped by a 532 nm, 10 W, diode-pumped solid-state laser (Tsunami and Millennia Xs, Spectra-Physics), generates a beam of ∼90 femtosecond, bandwidth limited pulses at 80 MHz, with approximately 1 W average power at a central wavelength of 910 nm. We used a group velocity dispersion pre-compensator with a pair of Brewster prisms in double-pass arrangements to introduce negative dispersion into the pulsed beam to correct the widening of pulse width caused by dispersive optics (1). The pulse width is measured by an autocorrelator (Carpe, APE). Laser beam is blocked by a computer controlled mechanical shutter (SH-10, Electro Optical Products Corporation) to the scanner and sample when not imaging. A rotating polygonal mirror and a galvanometric mirror form the scanning engine of the system. A 32-facet polygonal mirror (DT-32-394-035/LB12-5, Lincoln Laser) spins at approximately 22,000 rpm (revolutions per minute) to generate the fast-scan in the horizontal (X) direction. The horizontal scan rate is 11.733 kHz (85.23 µs period) and an active scan time of 59.3 µs (70%). The slower vertical (Y) scanning is achieved with a galvanometer driven mirror (VM2000 with 9.5 mm mirror, GSI Lumonics). When acquiring 512×512 pixel images, the frame rate is 22 fps with a pixel dwell time of 115 ns. The polygonal mirror may also be run at slower speeds (16,500 rpm, 11,000 rpm and 5,500 rpm) to increase pixel dwell times, if needed. The scanner is coupled to a custom epi-illumination fluorescence microscope based on commercial parts (Nikon). The Nikon objective lenses (Nikon: CFI 40XW and 60XW) are mounted to the nosepiece with a piezoelectric (PI: N-725 PIFOC) under computer control. The user can observe the sample in widefield mode through the eyepieces of a trinocular. To navigate on much larger sample area, the user can move the sample horizontally with a joystick that controls a motorized X-Y translation stage (BioPrecision 99S000, LUDL). The X-Y stage is mounted on a vertical stage (B49 Elevating Table, Velmex) that provides manual adjustment of Z position. During imaging, X-Y translation stage and piezoelectric (Z-axis) are controlled by the computer to perform manual or scripted automatic movement. After settling on the area of interest, the user engages the scanner coupling mirror to image in the multiphoton/confocal mode. The multi-photon fluorescence signal generated in the sample and collected by the objective lens is reflected towards the collection lens by a broadband, high OD, longpass dichroic mirror at 735 nm (FF735-Di01-25×36, Semrock). The collected fluorescence signal is passed through an array of dichroic filters, operating at 45 degrees, to bandpass emitter filters and onto the photomultiplier detectors (HC-120-13, Hamamatsu). The first beamsplitter has the center frequency of 520 nm (FF520-Di01-25×36, Semrock). The pass-through light is diverted by a secondary beamsplitter with a center frequency of 495 nm (FF495-Di01-25×36, Semrock), while the reflected light is diverted by a beamsplitter with a center frequency of 560 nm (FF560-Di01-25×36, Semrock). In this arrangement, the fluorescence signal is divided into four separate bands that are further filtered by bandpass elements before reaching the photomultipliers. Confocal detector module is placed at the entrance to the scanning system. It has a polarizing beamsplitter cube aligned with the beam in the P polarization for maximum transmission. A broadband quarter wave retarder (AQ-100-0840, Meadowlark Optics) is placed just before the objective lens at the far end of the optical path. The linearly polarized laser beam passes through the quarter wave retarder, positioned at 45 degrees to the incoming polarization, becomes circularly polarized, traverses the objective lens, and is focused on the sample. The light reflected back by the sample and collected by the objective lens passes the quarter wave retarder a second time to have a further 45 degrees phase added to induce a half-wave retardance and a 90-degree rotation of the linear polarization. On retracing the optical system, the polarizing beamsplitter cube will divert the sample-reflected light in to the confocal detector. Thus, all back-reflections from elements in the optical path before the quarter wave plate (that do not undergo a 90 degree polarization rotation) will be transmitted by the beamsplitter cube (onto the GVD and laser), and only reflections from the sample (and objective lens, unfortunately), that undergo a 90 degree rotation, will be directed to the confocal detector (2). Supplementary Figure S3 shows the functional schematic diagram of the connections and data flow of the control and acquisition system. We designed the timing synchronization electronics to reduce pixel jitter caused by timing mismatch between the free-running polygon mirror and the pixel clock of frame grabber. We used a diode laser and photodiode pair reflected off the polygonal mirror to generate the start of scan signal (SOS), which is conditioned by custom circuitry to generate the pixel clock phase matched to the rotating polygon. H-Sync and V-Sync signals that are also triggered by SOS, along with the pixel clock, are fed into the frame grabbers (PXI-1409, National Instruments) as the timing signals. Frame grabbers digitize the analog signals from the fluorescence and reflectance detectors to form the images. Thus, because imaging acquisition of fluorescence and reflectance signals is precisely synchronized with the free spinning polygon mirror, pixel jitter along the scanning lines is minimized to approximate 1/60 of pixel time. The control and acquisition hardware consists of a Windows PC workstation, DAC (Digital-Analog converter, PXI-6713 and PXI-6070, National Instruments) and video frame grabbers installed in the NI PXI-1000 external enclosure. Every frame grabber can take up to 4 inputs secuentially and digitizes the analog video signals into the images that later are retrieved by the host PC. Our system can acquire simultaneously from 3 video channels without dropping frames. Custom software written in Labview controls the majority of peripheral devices (laser power, PMT gain, Z focus, etc.) within the user interface. Captured images are pseudo-color coded and displayed in real time. We designed a series of imaging modes (single frame, multi-frame acquisition, time lapse, vertical Z-stack, X-Y mosacing, and scripted combinations of all above.) to meet various experimental needs. Acquired images are saved as TIFF files. We have also developed a series of software tools in Matlab (Mathworks Inc) to batch-process the stitching together of mosaiced images, projection of stacked images, etc. Further details of hardware and software can be obtained from authors. 1) O. E. Martinez, J. P. Gordon, and R. L. Fork, “Negative group-velocity dispersion using refraction,” J. Opt. Soc. Am. A 1, 1003–1006 (1984) 2) Pawley JB, Amos WB, Dixon A, Brelje TC: Simultaneous, non interfering, collection of optimal fluorescent and backscaltered light signals on the MRC-500/600. Proc. Microsc. Soc. Am. 51∶156–157 1993.
(PDF)

Figure S3 **YFP-B16 growth, immune infiltration and Pmel-1 T cell target potential is consistent with parental B16.** (A) 2.5×10^5^ B16 or YFP-B16 tumor cells were suspended in matrigel and inoculated into WT C57B/6 mice, 10 days later tumors were isolated, weighed (left panel) and a single cell suspension was prepared to asses for CD8+ T cell infiltration (middle panel). Representative FACS plots are shown (right panel). (B) Pmel-1 T cells (effectors), activated by xenogeneic human gp100 DNA vaccination were co-cultured for 4 hours with either YFP-B16 or its parental B16 (targets). After 4 hours, co-cultures were labeled with CD8 and 7AAD and specific lysis of tumor cells was calculated using the number of CD8− 7AAD+/7AAD− in each well, normalized to tumor cells alone.
(PDF)

Figure S4 **T cells are localized in the vicinity of blood vessels in B16 tumors and tumor cell death is not detected in regions infiltrated by Pmel-1 T cells.** 1×10^5^ YFP-B16 tumor cells were suspended in matrigel and inoculated into WT C57B/6 mice, and 3 days later 3×10^5^ naive CD8+ GFP or CFP Pmel-1 T cells were AT (N = 4). After 10 days, mice were used for intravital TPLSM after which tumors were excised maintaining orientation, and noting location of site imaged during TPLSM. Fresh frozen sections were prepared and underwent either H&E or immuno-fluorescence staining. (A) Representative 20X H&E staining of a day 10 YFP-B16 tumor, insets are 40X views with black arrows pointing to positions of leukocytes. For reference, scale bars = ∼100 µm for 20x, and ∼50 µm for 40X (B) Two representative 20X immuno-fluorescence sections with CD31 (magenta) overlaid with CD8 (green) and DAPI (blue). White arrows point to regions of concentrated CD8 label which coincides with CD31 labeling. Approximate scale bars are shown = 100 µm for 20x, and ∼50 µm for 40X (C) Intra-tumor T cells positions were calculated by producing a high digital threshold map of the tumor images (left) and then comparing Volocity calculated cell centroid positions with tumor map to determine cell location with respect to tumor or “not tumor” using Matlab. Points represent intra-tumor location vs. instantaneous speed for each cell tracked, for a single time point. (D) 1×10^5^ YFP-B16 (green) tumor cells were suspended in Matrigel and inoculated into WT C57B/6 mice with AT 3 days later of 3×10^5^ naive CD8+ CFP-Pmel-1 T cells. Tumors were imaged 7 days after CFP-Pmel-1 transfer. After imaging CFP-Pmel-1 mobility, 1 mg/ml of propidium iodide was directly injected into the center of the tumor (magenta) while the mouse was maintained under intravital imaging conditions. Reflectance (blue) is show in the left panels to demonstrate the outside edge of the tumor. Propidium iodide staining is restricted to the regions on the outside edge of the tumor only (center panel) and there is no staining inside the tumor mass. As a positive control (right panel), mice with YFP-B16 tumors were treated with chemotherapy (250 µg/kg of cyclophosphamide) 6 days after tumor challenge and then imaged 4 days later. In the chemotherapy treated example, PI staining is seen deep into the tumor mass, staining larger nuclei in areas of faint YFP label.
(PDF)

Movie S1 **GFP-Pmel-1 T cells traffic through TDLN High endothelial venules** **(HEV).** 30×10^6^ GFP-Pmel-1 T cells were transferred into a YFP-B16 tumor bearing mouse. One hr after transfer, TDLN was imaged at a single plane. Reflectance is shown in blue, GFP-Pmel-1 in red, HEV are outlined. Movie is in real time, 20fps. Blood flow and red blood cells can be seen moving in the HEV using the reflectance channel. A GFP-Pmel-1 T cell can immediately be seen adhering to the vessel wall. After 1 second a GFP-Pmel-1 T cell, (1st white arrow) and after 12 seconds an unlabeled cell in reflectance (2nd white arrow) can be seen rolling in the HEV.
(AVI)

Movie S2 **Unlabeled cells appear as YFP-Ghosts moving through YFP-tumor.** Birds eye 3D movie of single quadrant from a YFP-B16 tumor (green). Unlabeled YFP-Ghosts (dark voids in the YFP signal can be seen moving throughout the tumor during imaging. Tumor regions were considered viable if YFP-ghosts could be seen migrating in the region imaged. 132 seconds separate each frame.
(AVI)

Movie S3 **Pmel-1 T cells engage in long term interactions with YFP-B16 tumor cells.** Representative composite time-lapse movies zoomed into two regions of mosaic movie S3. GFP-Pmel-1 in red, YFP-B16 in green, tracks are shown in blue. Left panel and right panels are close up images of annotated cells from the bottom left of and bottom right of movie S3, respectively. Pmel-1 T cells can be seen engaging in long term interactions with tumor cells (white arrows) throughout the time period of the image. Black arrow points to a Pmel-1 T cell which engages a tumor cell for approximately 20 min before disengaging and moving away. One hundred and fifteen seconds separate each frame.
(AVI)

Movie S4 **OT-1 T cells infiltrate the same region of YFP-B16 tumor and are more mobile than Pmel1-T cells.** Large view representative time-lapse image of YFP-B16 (green) tumor 7 days post transfer of 3×10^5^ naive CD8+ CFP-Pmel-1 T cells (Cyan) and CD8+ GFP-OT-1 (red) T cells. Birds eye 3D image contains 6 adjacent regions. Seventy-seven seconds separate each frame. Global view of tumor shows GFP-OT-1 T cells moving faster and less confined than CFP-Pmel-1 T cells.
(AVI)

Movie S5 **Non-specific OT-1 T cells do not engage in long term interactions with B16 tumor cells.** Representative time-lapse image shows close up view of the top middle region from movie S6. YFP-B16 (green) tumor 7 days post transfer of 3×10^5^ naive CD8+ CFP-Pmel-1(Cyan) and CD8+ GFP-OT-1 (red) T cells. Representative T cell tracks are show in red for OT-1 T cells, and blue for Pmel-1 T cells. Density of YFP in the image has been reduced to permit better observation of OT-1 and Pmel-1 T cells. Seventy-seven seconds separate each frame. Arrows (white = Pmel-1, black = OT-1) point to examples of cells which are engaged with tumor cells at the beginning of the movie. Movie demonstrates that non-specific GFP-OT-1 T cells do not engage in long term interactions with tumor cells in contrast to tumor-specific CFP-Pmel-1 T cells.
(AVI)

Movie S6 **OT-1 T cells have reduced mobility and engage in long term interactions with ova expressing MO4-YFP tumor cells.** Representative time-lapse image shows MO4-YFP (green) tumor 7 days post transfer of 3×10^5^ naive CD8+ GFP-OT-1 (red) T cells. Representative T cell tracks are show in red. Movie demonstrates that in OVA expressing MO4-YFP, GFP-OT-1 T cells have mobility and interact with tumor cells similar to Pmel-1 T cells.
(AVI)
