# Supplementary figures and images for: Detection of Intra-Tumor Self Antigen Recognition during Melanoma Tumor Progression in Mice Using Advanced Multimode Confocal/Two Photon Microscope
Source: PLoS One. 2011 Jun 22;6(6):e21214. doi: 10.1371/journal.pone.0021214 (PMC3120835; doi:10.1371/journal.pone.0021214)

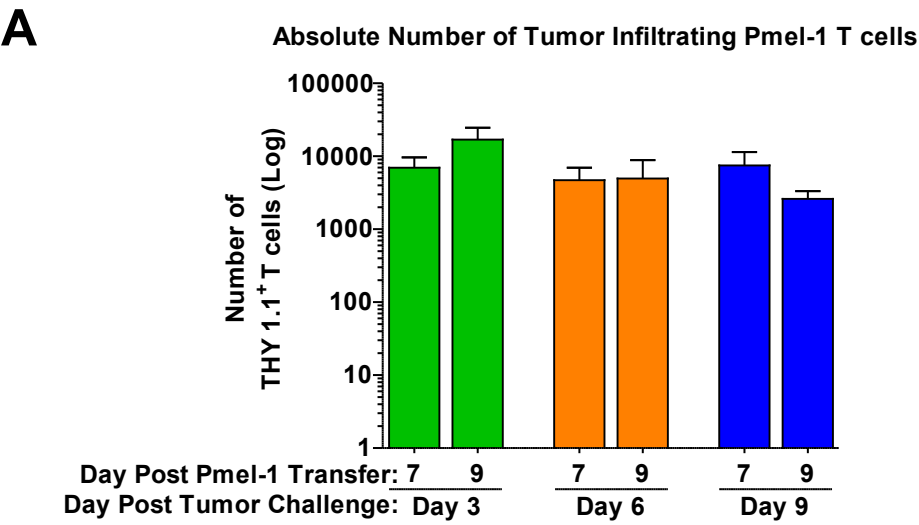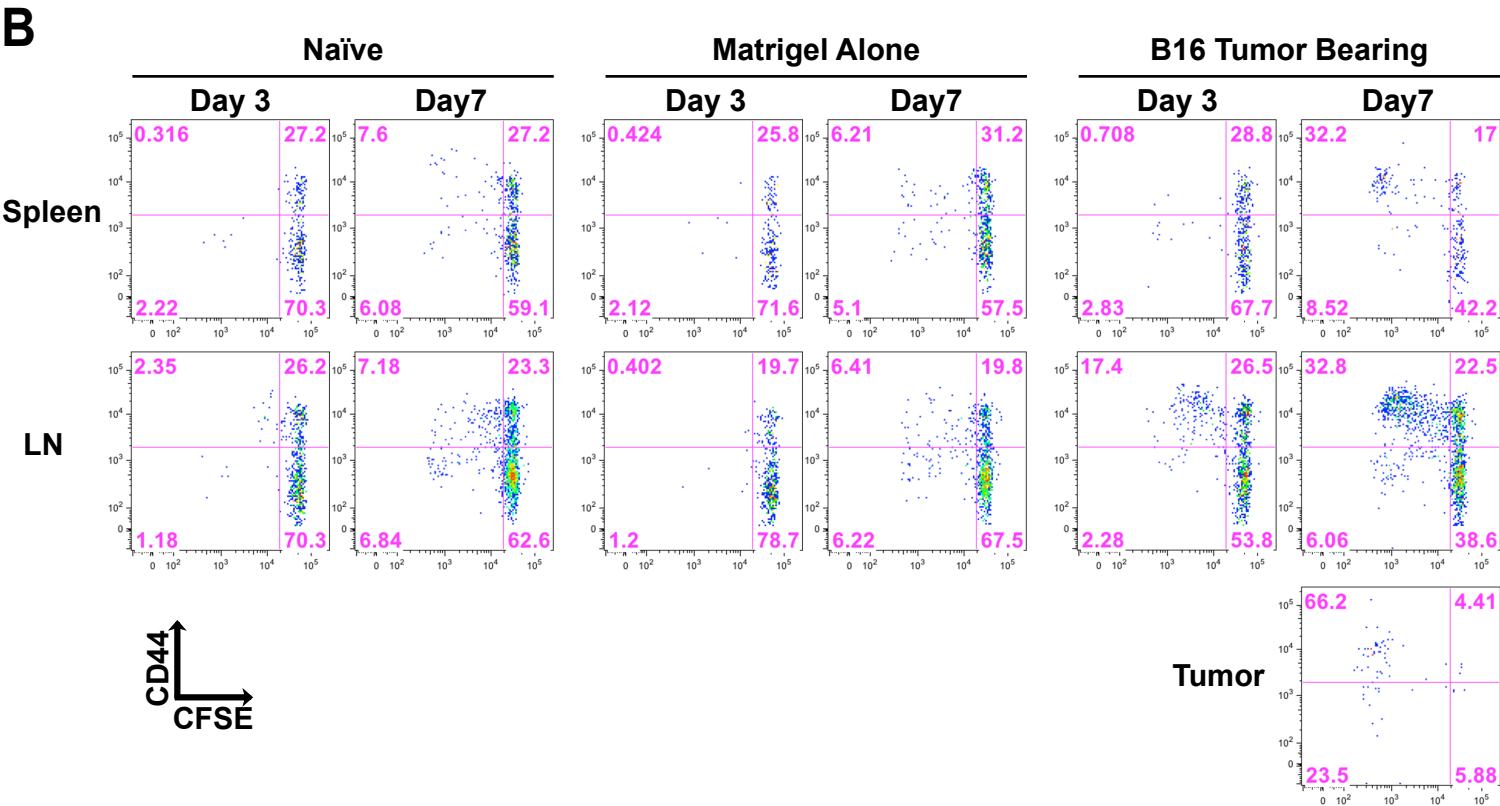

Supplement: Figure S1 — Adoptively transferred Pmel-1 T cells do not prime in the absence of tumor. A) 3×10^5^ Naive CFSE labeled Thy 1.1+ CD8+ Pmel-1 T cells were AT into B16 tumor bearing C57BL/6 mice 3, 6 or 9 day after tumor challenge. Tumors were assessed for Pmel-1 T cell infiltration 7 and 9 days after transfer, corresponding with day 10–12, 13–15, and 16–18 after tumor challenge for the three groups. No significant differences were seen in the absolute number of Pmel-1 T cells infiltrating the tumor when comparing all groups (One way anova, tukey multiple comparison). B)_1×106 Naive CFSE labeled Thy 1.1+ CD8+ Pmel-1 T cells were AT into naïve C57BL/6 mice. Spleens and Lymph nodes were isolated and single cell suspensions were prepared from 3 mice every 48 hours beginning at 24 hours after transfer for up to 7 days. Samples were then analyzed to determine the extent of Pmel-1 T cell infiltration and the activation state of the infiltrating cells. (LEFT) SSC/Thy 1.1 FACS plots are representative from 7 days post transfer. Percentages of tissue infiltrating Thy 1.1+ Pmel-1 T cells are shown in the gate. RIGHT panels show CD44/CFSE on noted days post transfer in Spleen (top) and Lymph node (bottom). Gates show representative percentages of activated (CD44High/CFSELow) Pmel-1 T cells infiltrating tissues at each time point from the parent SSClow Thy 1.1+gate. Example is representative of 2 independent experiments. (PDF) [file pone.0021214.s001.pdf]

Sup Figure 2

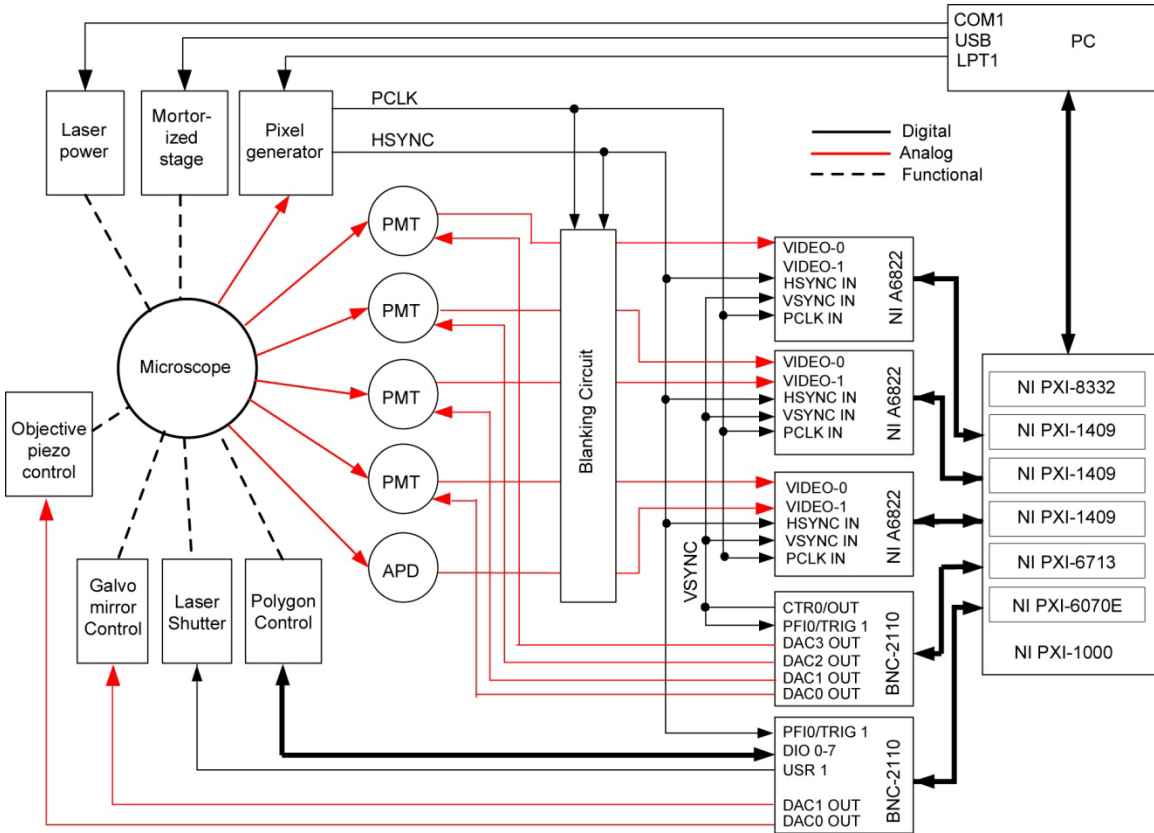

Supplement: Figure S2 — Schematic Blueprints for Dual mode cofocal/two photon microscope. Complete list of abbreviations form figure 3A: (ac) autocorrelator, (at) attenuator, (ccd) charge-coupled device, (cl) collection lens, (ei) Epifluorescence illumination, (fds) fluorescence detection system, (gc) group-velocity dispersion compensator, (pl) pump laser, (qwp) quarter-wave plate, (sl) synchronization diode laser, (sp) synchronization photodetector (to) trinocular. As illustrated in Figure 3A, a mode locked titanium-sapphire femtosecond laser resonator, pumped by a 532 nm, 10 W, diode-pumped solid-state laser (Tsunami and Millennia Xs, Spectra-Physics), generates a beam of ∼90 femtosecond, bandwidth limited pulses at 80 MHz, with approximately 1 W average power at a central wavelength of 910 nm. We used a group velocity dispersion pre-compensator with a pair of Brewster prisms in double-pass arrangements to introduce negative dispersion into the pulsed beam to correct the widening of pulse width caused by dispersive optics (1). The pulse width is measured by an autocorrelator (Carpe, APE). Laser beam is blocked by a computer controlled mechanical shutter (SH-10, Electro Optical Products Corporation) to the scanner and sample when not imaging. A rotating polygonal mirror and a galvanometric mirror form the scanning engine of the system. A 32-facet polygonal mirror (DT-32-394-035/LB12-5, Lincoln Laser) spins at approximately 22,000 rpm (revolutions per minute) to generate the fast-scan in the horizontal (X) direction. The horizontal scan rate is 11.733 kHz (85.23 µs period) and an active scan time of 59.3 µs (70%). The slower vertical (Y) scanning is achieved with a galvanometer driven mirror (VM2000 with 9.5 mm mirror, GSI Lumonics). When acquiring 512×512 pixel images, the frame rate is 22 fps with a pixel dwell time of 115 ns. The polygonal mirror may also be run at slower speeds (16,500 rpm, 11,000 rpm and 5,500 rpm) to increase pixel dwell times, if needed. The scanner is coupled to a [file pone.0021214.s002.pdf]

Sup Figure 3

**A**

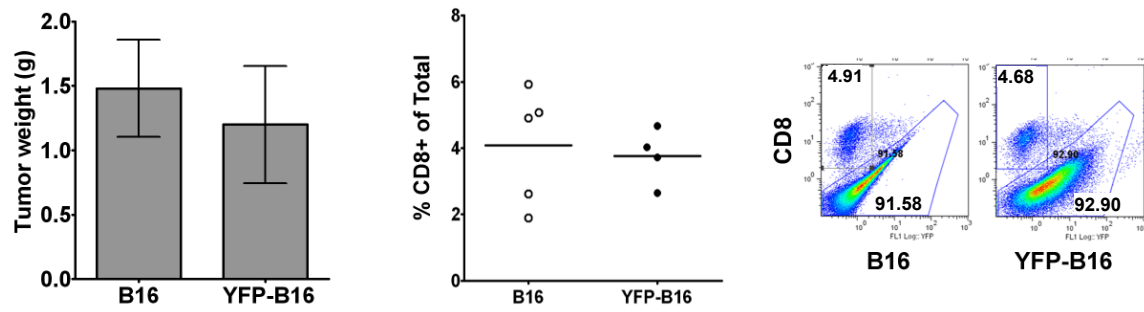

**B**

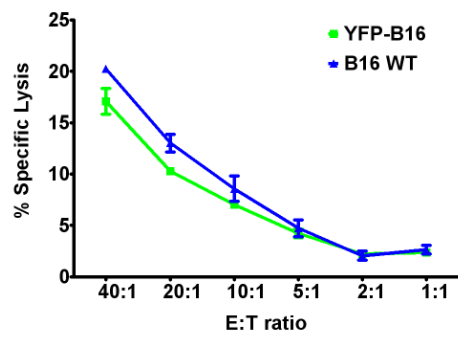

Supplement: Figure S3 — YFP-B16 growth, immune infiltration and Pmel-1 T cell target potential is consistent with parental B16. (A) 2.5×10^5^ B16 or YFP-B16 tumor cells were suspended in matrigel and inoculated into WT C57B/6 mice, 10 days later tumors were isolated, weighed (left panel) and a single cell suspension was prepared to asses for CD8+ T cell infiltration (middle panel). Representative FACS plots are shown (right panel). (B) Pmel-1 T cells (effectors), activated by xenogeneic human gp100 DNA vaccination were co-cultured for 4 hours with either YFP-B16 or its parental B16 (targets). After 4 hours, co-cultures were labeled with CD8 and 7AAD and specific lysis of tumor cells was calculated using the number of CD8− 7AAD+/7AAD− in each well, normalized to tumor cells alone. (PDF) [file pone.0021214.s003.pdf]

**A**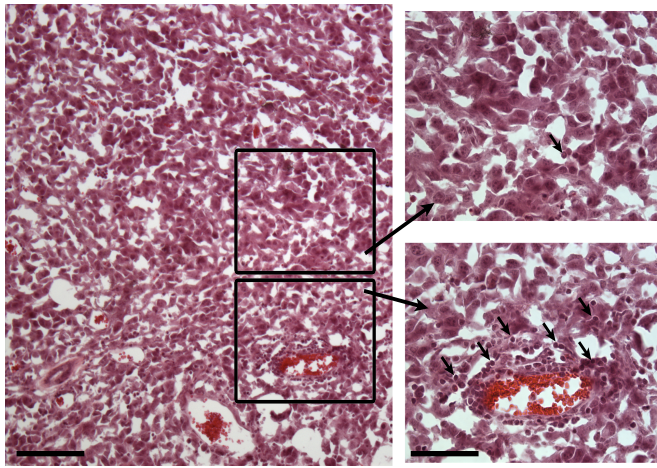**B**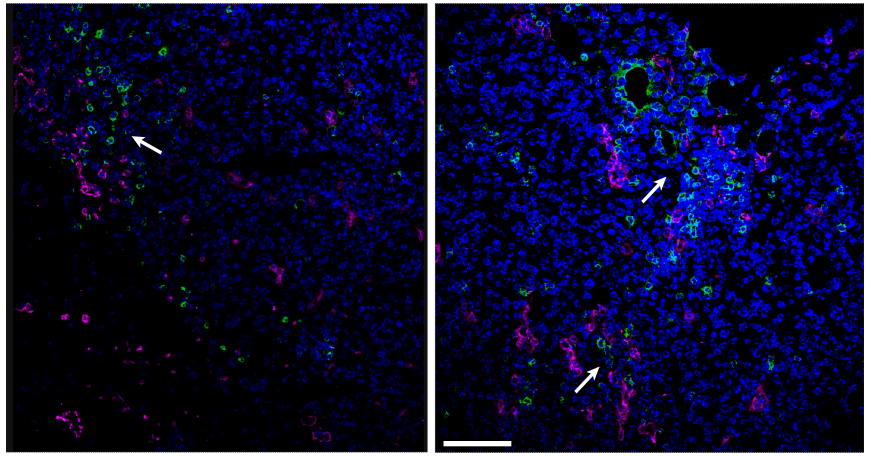**C**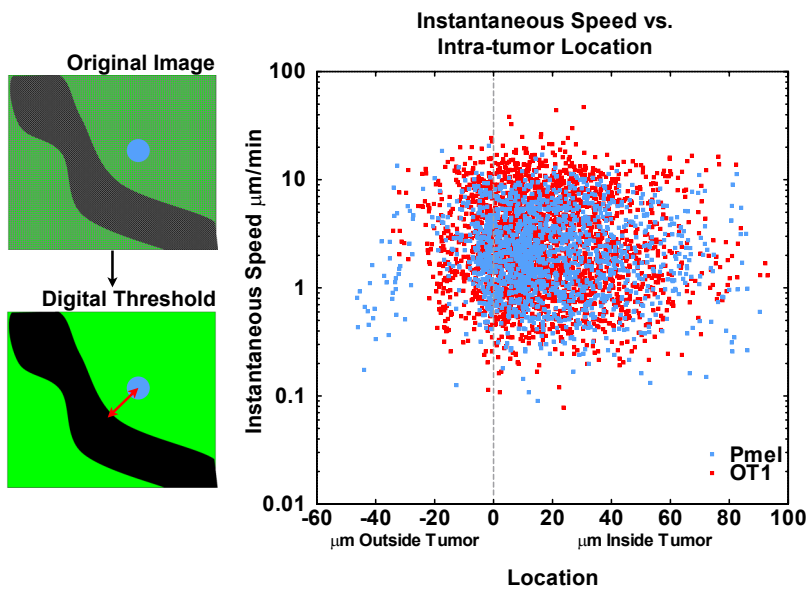**D**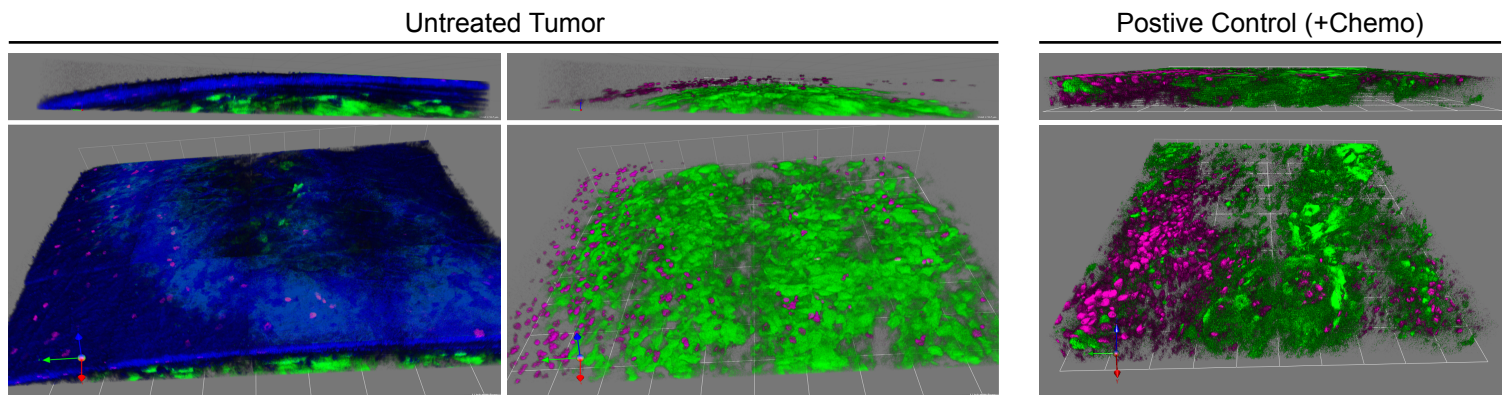

Supplement: Figure S4 — T cells are localized in the vicinity of blood vessels in B16 tumors and tumor cell death is not detected in regions infiltrated by Pmel-1 T cells. 1×10^5^ YFP-B16 tumor cells were suspended in matrigel and inoculated into WT C57B/6 mice, and 3 days later 3×10^5^ naive CD8+ GFP or CFP Pmel-1 T cells were AT (N = 4). After 10 days, mice were used for intravital TPLSM after which tumors were excised maintaining orientation, and noting location of site imaged during TPLSM. Fresh frozen sections were prepared and underwent either H&E or immuno-fluorescence staining. (A) Representative 20X H&E staining of a day 10 YFP-B16 tumor, insets are 40X views with black arrows pointing to positions of leukocytes. For reference, scale bars = ∼100 µm for 20x, and ∼50 µm for 40X (B) Two representative 20X immuno-fluorescence sections with CD31 (magenta) overlaid with CD8 (green) and DAPI (blue). White arrows point to regions of concentrated CD8 label which coincides with CD31 labeling. Approximate scale bars are shown = 100 µm for 20x, and ∼50 µm for 40X (C) Intra-tumor T cells positions were calculated by producing a high digital threshold map of the tumor images (left) and then comparing Volocity calculated cell centroid positions with tumor map to determine cell location with respect to tumor or “not tumor” using Matlab. Points represent intra-tumor location vs. instantaneous speed for each cell tracked, for a single time point. (D) 1×10^5^ YFP-B16 (green) tumor cells were suspended in Matrigel and inoculated into WT C57B/6 mice with AT 3 days later of 3×10^5^ naive CD8+ CFP-Pmel-1 T cells. Tumors were imaged 7 days after CFP-Pmel-1 transfer. After imaging CFP-Pmel-1 mobility, 1 mg/ml of propidium iodide was directly injected into the center of the tumor (magenta) while the mouse was maintained under intravital imaging conditions. Reflectance (blue) is show in the left panels to demonstrate the outside edge of the tumor. Propidium iodide staining is restricted to the regions on the outsid [file pone.0021214.s004.pdf]
